# Cordycepin, an Active Constituent of Nutrient Powerhouse and Potential Medicinal Mushroom *Cordyceps militaris* Linn., Ameliorates Age-Related Testicular Dysfunction in Rats

**DOI:** 10.3390/nu11040906

**Published:** 2019-04-23

**Authors:** Spandana Rajendra Kopalli, Kyu-Min Cha, Sang-Ho Lee, Seock-Yeon Hwang, Young-Joo Lee, Sushruta Koppula, Si-Kwan Kim

**Affiliations:** 1 Department of Bioscience and Biotechnology, Sejong University, Gwangjin-gu, Seoul 05006, Korea; spandanakopalli@gmail.com (S.R.K.); yjlee@sejong.ac.kr (Y.-J.L.); 2 Department of Integrated Biosciences, College of Biomedical & Health Science, Konkuk University, Chungju 27381, Korea; chakm89@kku.ac.kr (K.-M.C.); lyswo3@kku.ac.kr (S.-H.L.); koppula@kku.ac.kr (S.K.); 3 Department of Biomedical Laboratory Science, College of Applied Science and Industry, Daejeon University, Daejeon 300-716, Korea; syhwang@dju.kr

**Keywords:** Cordycepin, antioxidant, sex hormone receptors, spermatogenesis, SIRT1, mTORC1

## Abstract

Age-related male sexual dysfunction covers a wide variety of issues, together with spermatogenic and testicular impairment. In the present work, the effects of cordycepin (COR), an active constituent of a nutrient powerhouse *Cordyceps militaris* Linn, on senile testicular dysfunction in rats was investigated. The sperm kinematics, antioxidant enzymes, spermatogenic factors, sex hormone receptors, histone deacetylating sirtuin 1 (SIRT1), and autophagy-related mammalian target of rapamycin complex 1 (mTORC1) expression in aged rat testes were evaluated. Sprague Dawley rats were divided into young control (2-month-old; YC), aged control (12-month-old; AC), and aged plus COR-treated groups (5 (COR-5), 10 (COR-10), and 20 (COR-20) mg/kg). The AC group showed reduced sperm kinematics and altered testicular histomorphology compared with the YC group (*p* < 0.05). However, compared with the AC group, the COR-treated group exhibited improved sperm motility, progressiveness, and average path/straight line velocity (*p* < 0.05–0.01). Alterations in spermatogenesis-related protein and mRNA expression were significantly ameliorated (*p* < 0.05) in the COR-20 group compared with the AC group. The altered histone deacetylating SIRT1 and autophagy-related mTORC1 molecular expression in aged rats were restored in the COR-20 group (*p* < 0.05). In conclusion, the results suggest that COR holds immense nutritional potential and therapeutic value in ameliorating age-related male sexual dysfunctions.

## 1. Introduction

As men age, decreased testicular function and deterioration of sexual function, accompanied by mood changes, low libido, or physical transformations, commonly known as “andropause”, is observed [1]. Typical physiological changes in aging men include sexual organ atrophy, delay in achieving full penile erection, poor quality of erection, and decline in orgasm intensity [2,3]. Additionally, age-dependent changes are observed in the testes, both in the rate of steroidogenesis and spermatogenesis [4].

Male reproductive function depends on the intermittent secretion of luteinizing hormone (LH) and follicle-stimulating hormone (FSH) by the pituitary gland under the influence of gonadotropin-releasing hormone [5]. LH stimulates testicular Leydig cells to secrete testosterone, with a negative feedback mechanism to the hypothalamus to modulate LH secretion [6]. FSH stimulates testicular Sertoli cells and promotes spermatogenesis. 

Endocrine dysfunction in young males with pituitary deficiency, also known as Klinefelter’s syndrome, is primarily attributed to defects at the testicular region or defects in the regulation of hypothalamus/pituitary gland [7]. However, changes in the regulation and progression of the hypothalamic–pituitary–gonadal axis, which is associated with decreased serum testosterone levels, might be one of the characteristic features of reproductive dysfunction in aging men [7]. Furthermore, aging is also associated with increased free radicals and generation of other reactive species with decreased antioxidative defenses, resulting in chronic oxidative stress [8,9].

Although aging is an inevitable process, delaying the progressive degenerative effects of aging, particularly in the testes, may help control male sexual dysfunctions (MSDs). Several nutritional and traditional herbs, such as *Panax ginseng*, *Withania somnifera*, *Pausinystalia johimbe*, and *Erycoma longifolia*, have been used for a considerable period of time in many parts of the world, including Asia, to treat MSD and shown potential in enhancing testicular functions disrupted by aging [10,11,12]. These natural agents are attractive because they provide health benefits beyond those related to MSDs and are inexpensive compared to prescription medications. The basis of these nutrient herbal therapies used for MSD is that they help aging males improve their ability to overcome sexual dysfunction by increasing sexual stimulation as well as erectile, ejaculatory, orgasmic, and other responses [13,14]. Most of these herbal nutrients have immense antioxidant, anti-inflammatory, and immunomodulatory properties, which helps regulate the altered oxidative defense system, spermatogenesis-related factors, and production of sex hormones. 

*Cordyceps militaris* Linn. (family, Cordycipitaceae; *C. militaris*) is a valuable nutrient powerhouse and traditional medicinal mushroom that is well known since antiquity for its ability to revitalize various organ systems [15]. As one of the earliest known natural remedy, *C. militaris* is used extensively as a crude medicament and food in Asian countries [16]. Pharmacologically, *C. militaris* possesses antioxidant, anti-inflammatory, antiaging, anticancer, antiproliferative, antimetastatic, immunomodulatory, antimicrobial, antifibrotic, steroidogenic, hypoglycemic, hypolipidemic, antiangiogenetic, antidiabetic, neuroprotective, renoprotective, and pneumo-protective properties [17,18,19]. Among the components of *C. militaris*, cordycepin, also known as 3-deoxyadenosine, a purine nucleoside derivative, is a well-studied active constituent with significant biological properties, such as antitumor, antiviral, anti-inflammatory, and antiatherosclerotic effects [20,21]. Several studies have demonstrated that cordycepin inhibits platelet aggregation [22], induces apoptosis [23], prevents hyperglycemia [24], and inhibits the migration/proliferation of vascular smooth muscle cells and vascular neointimal formation [25]. Cordycepin is also known to enhance sexual function in males [21]. Structurally, cordycepin differs from adenosine by the absence of oxygen at the 3′ position of its ribose moiety. However, because of its structural similarity with adenosine, certain enzymes cannot discriminate between the two molecules. Therefore, cordycepin is readily phosphorylated intracellularly, enabling its participation in several physiological and biochemical reactions.

Previous studies have suggested that *C. militaris* improves sexual function, supports the treatment of erectile dysfunction (ED), and acts as a pro-sexual agent. *C. militaris* supplementation has also been found to improve sperm quality and quantity in rats [26]. Previous reports from our laboratory showed that cordycepin attenuated age-related oxidative stress and ameliorated antioxidant capacity in old rats [27]. However, the beneficial role of cordycepin and its modes of action in age-related testicular dysfunction remain unclear. In the present study, we investigated the ameliorative effects of cordycepin on age-related changes in sperm kinematics and expression of spermatogenesis-related key biomolecules, such as sex hormone receptors, oxidation-regulating enzymes, transcription factors as well as histone deacetylating sirtuin 1 (SIRT1) and autophagy-related mammalian target of rapamycin complex 1 (mTORC1) molecular changes in aged rats.

## 2. Materials and Methods 

### 2.1. Isolation and Identification of Cordycepin from C. militaris

Cordycepin from *C. militaris* was isolated as described in our previous report [27]. Briefly, *C. militaris* mycelia were extracted three times using 70% methanol for 4 h with reflux. The obtained methanol extract was then evaporated under reduced pressure and partitioned three times between butanol and water. The butanol fractions were pooled, dried in vacuo, and subjected to silica column chromatography (chloroform ethyl acetate: methanol 12:1:3, v/v). The cordycepin-rich fraction was recrystallized in absolute ethanol to obtain pure cordycepin (hereafter referred to as COR) identified by ^1^H (500 MHz) and ^13^C (125 MHz) nuclear magnetic resonance (JEOL, Tokyo, Japan). The purity of COR was determined by melting point analysis (224–226 °C; Manstead/electrothermal melting point analyzer, IA9100), optical rotation (JASCO P-1020 polarimeter, Tokyo, Japan; filter 589 mm, cylindrical glass cell 10 × 100 mm, −49.39 in methanol), and reverse-phase (C18, 4.6 × 250 mm, 5 µm, 13% aqueous methanol, 260 nm) and normal-phase high-pressure liquid chromatography (silica gel, 4.6 × 250 mm, 5 µm, CHCl_3_: methanol: 5:5, 260 nm).

### 2.2. Experimental Animals and Design

Fifty male Sprague Dawley rats (40 12-month-old 750 ± 20 g and 10 2-month-old 280 ± 10 g) were purchased from Hanil Experimental Animal Breeding Co. Ltd. (Yeumsung, Chungbuk, Korea) and acclimatized to the Regional Innovation Center Experimental Animal Facility, Konkuk University, Republic of Korea, for at least 1 week prior to the experiment. Rats were provided a standard pellet diet and water ad libitum and maintained at a constant temperature (23 ± 2 °C) and relative humidity (55 ± 10%) on a 12/12 h light/dark cycle. Rats were divided into five groups (*n* = 10): the young control (YC) and aged control (AC) groups received vehicle (distilled water) only, whereas the COR-treated groups were administrated COR at daily doses of 5 mg/kg body weight (b.w.) (COR-5), 10 mg/kg b.w. (COR-10), and 20 mg/kg b.w. (COR-20) for 6 months. The COR doses were selected based on our previously reported study [27]. COR was mixed homogenously with the sterilized standard diet and administered orally after pelletization. The defined daily dose of COR was managed by preparing the food freshly every two weeks considering the weight of the rats every week and calculating their daily dietary intake. After the experiment, the animals were fasted for 24 h (only water was provided ad libitum), followed by sacrifice with carbon dioxide according to National Institutes of Health (NIH) guidelines. The YC group was 8 months old and AC/COR groups were 18 months old at the time of sacrifice. All animal experiments were performed in accordance with the Institutional Animal Care and Use Committee guidelines of Konkuk University, and the study was approved by the Animal Ethics Committee in accordance with the 14^th^ article of the Korean Animal Protection Law. The epididymis and testes were excised and washed in ice-cold saline, and the adhering fat and connective tissues were cleaned. A 10% homogenate of the testis tissue was prepared in Tris–HCl buffer (0.1 M, pH 7.4), centrifuged (2500 rpm for 10 min at 4°C) to pellet the cell debris, and the clear supernatant was used for Western blotting and other biochemical assays. 

### 2.3. Sperm Kinematic Studies

Sperm samples were obtained by extracting the sperm cells from the left caudal epididymis after excising with scissors. A computer-assisted sperm analyzer (CASA, Hamilton Thorne Inc., Beverly, MA, USA) equipped with a 4× objective lens and charge-coupled device camera was used to record sperm kinematic parameters as described previously [14]. 

### 2.4. Histology and Analysis of Spermatogenesis-Related Parameters

For histopathological analysis, the left testis was cut into small pieces (3–5 mm^3^) and fixed in Bouin’s solution (saturated solution of picric acid, 40% formaldehyde, and glacial acetic acid). Fixation of the testicular tissue, horizontal sectioning of the tissue, staining of the specimen, observation of the seminiferous tubules, analysis of stages of spermatogenesis, and categorization of the criteria were performed as described in our previous report [14]. 

### 2.5. Western Blot Analysis 

The procedure for protein expression analysis was followed by Western blotting technique as described earlier [14]. Briefly, testis protein from each samples were separated by 10% sodium dodecyl sulfate–polyacrylamide gel electrophoresis and transferred to polyvinylidene fluoride membranes (Millipore, Billerica, MA, USA). Each membrane was incubated for 1 h in Tris-buffered saline containing 0.1% Tween-20 and 5% skim milk to block nonspecific binding. The membranes were then incubated with specific primary antibodies (1:1000 dilution; Santa Cruz Biotechnology, Dallas, TX, USA). The internal control used was β-Actin. The proteins were detected using horseradish peroxidase-conjugated secondary antibodies and a chemiluminescence detection system (GE Healthcare Life Sciences, Little Chalfont, UK). 

### 2.6. Reverse Transcription–Polymerase Chain Reaction (RT–PCR)

RNABee reagent (AMS Bio, Abingdon, UK) was used to extract the total RNA according to the manufacturer's instructions, and 1 μg of extracted RNA was reverse-transcribed as previously described [28]. Polymerase chain reaction (PCR) was performed as described in our previous report [14], and the primers used for the study are shown in Table 1. Band intensities were normalized to glyceraldehyde-3-phosphate dehydrogenase (GAPDH) band analyzed by NIH ImageJ software (version 1.41o; National Institutes of Health, Bethesda, MD, USA). 

### 2.7. Statistical Analysis

The results are expressed as the mean ± standard error of the mean (SEM; *n* = 10). Statistical significance was analyzed by Student's *t*-test for comparisons between two groups and analysis of variance (ANOVA) for multiple comparisons using the GraphPad Prism version 4.0 (GraphPad, Inc., San Diego, CA, USA). Values of *p* < 0.05 were considered statistically significant.

## 3. Results

### 3.1. Interpretation of Cordycepin Structure

**Figure 1:**
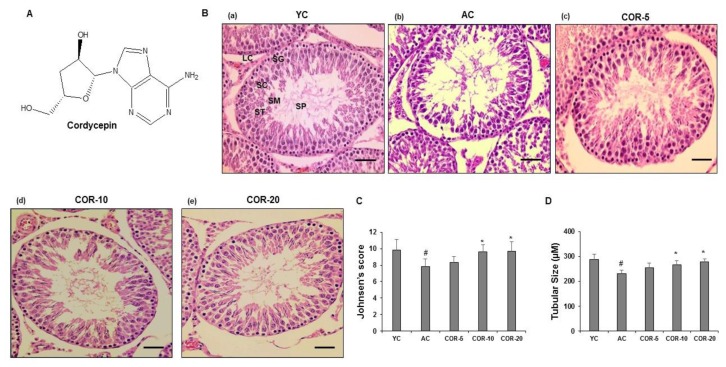
Chemical structure, histological analysis, Johnsen’s scores, and seminiferous tubule size in aged rat testes. (**A**) Cordycepin structure. (**B**) Representative images of tubular cross sections of testis: (**a**) young control rats YC, (**b**) vehicle-treated aged rats AC, (**c**) COR 5 mg/kg treated aged rats, (**d**) COR 10 mg/kg treated aged rats, and (**e**) COR 20 mg/kg treated aged rats. Sections were stained with hematoxylin and eosin (H&E). The images are typical of those obtained in five independent experiments. Scale bar = 45 μM and magnification = 200x. (**C**) Johnsen’s score. (**D**) Tubular size (µM). The results are expressed as mean ± SEM (*n* = 10). ^#^ *p* < 0.05 compared with YC group and ^*^ *p* < 0.05 compared with AC group. COR, cordycepin; LC, Leydig cell; ST, Sertoli cell; SG, spermatogonia; SC, spermatocyte; SM, spermatid; SP, spermatozoa.

The structure of cordycepin (COR) is shown in Figure 1A. The isolated COR was an amorphous white powder with a purity of 99.99% as determined by HPLC. The HPLC chromatogram is shown in Supplementary Material Figure S1.

### 3.2. Effect of COR on Sperm Kinematics and Testis Morphology 

The sperm motility ratios in the YC and AC groups were 74.7 ± 8.8% and 34.0 ± 8.7%, respectively (*p* < 0.05). The ratios of sperm progressiveness were 33.7 ± 4.2% and 12.7 ± 3.9% in YC and AC rats, respectively (*p* < 0.05). Overall, aging considerably attenuated sperm quality. Particularly, the average path velocity (VAP), straight line velocity (VSL), linearity (LIN), and wobble of sperm swimming (WOB) of AC rats showed markedly lower values compared with the YC rats. However, curvilinear velocity (VCL) and straightness of sperms were similar in YC and OC rats. Additionally, high doses of COR (COR-10 and COR-20) significantly improved (*p* < 0.05 and *p* < 0.01) specific parameters associated with sperm quality, such as motility, progressiveness, VAP, VSL, LIN, and WOB values. The data with their statistical significance of each group is shown in Table 2. 

The results of histopathological examination are shown in Figure 1B. The saline-treated YC group showed a normal pattern (a). Germ cells juxtaposed to the basal membrane inward to the lumen of the tubule, reflecting the chronological arrangement of maturing stage. However, several changes were observed in the testes of the AC group; for example, the arrangement of cells in the seminiferous tubules was loose and irregular, with shedding of cellular material from the seminiferous epithelium. The number of spermatogonia (stem cells that produce spermatocytes) was lower, and the basal layer of the germinal epithelium was degenerated in the AC group testes compared with in the YC group testes (b). In contrast, the COR-treated groups (5, 10, and 20 mg/kg) showed improvement at higher doses, such as densely packed spermatogonia and Sertoli cells rested on the basement membrane surrounded by a concentric myofibroblast layer. Additionally, Leydig cells were observed in the interstitial space between the seminiferous tubules in the COR-treated groups but not in the AC group (c–e).

### 3.3. Effect of COR on Spermatogenesis-Related Histological Parameters

**Figure 2:**
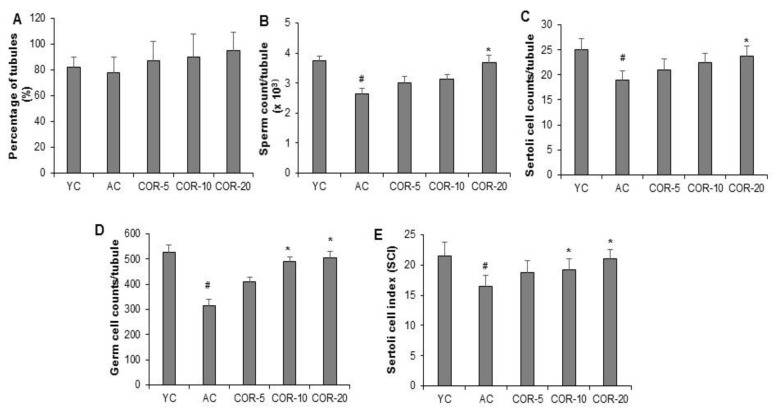
Effect of COR on the spermatogenesis parameters in aged rats. (**A**) Percentage of tubules with sperm, (**B**) sperm count per tubule, (**C**) Sertoli number, (**D**) germ cell count, and (**E**) Sertoli cell index. ^#^ *p* < 0.05 compared with YC group and ^*^ *p* < 0.05 compared with AC group. YC, young rats; AC, aged rats; COR, cordycepin.

Selected parameters related to spermatogenesis were evaluated using light microscopy. As shown in Figure 1C,D, aging significantly reduced the Johnsen’s score and seminiferous tubular size (*p* < 0.05). However, COR treatment significantly ameliorated these changes (*p* < 0.05). Furthermore, no significant difference in the percentage of seminiferous tubules with sperms was observed between the YC and AC groups (Figure 2A). However, sperm, Sertoli cell, and germ cell counts per tubule and SCI (Figure 2B–E) were significantly (*p* < 0.05) reduced in the AC group. Treatment of AC rats with 10 and 20 mg/kg COR significantly (*p* < 0.05) increased the levels of the above spermatogenesis-related factors. Based on the above results, further mechanistic studies of the expression levels of sex hormone receptors, antioxidative enzymes, key spermatogenesis-related factors, histone-deacetylating SIRT1, and autophagy-related mTORC1 were performed in aged rat testes treated with 20 mg/kg COR. 

### 3.4. Effect on Protein and mRNA Levels of Sex Hormone Receptors in Aged Rat Testes 

**Figure 3:**
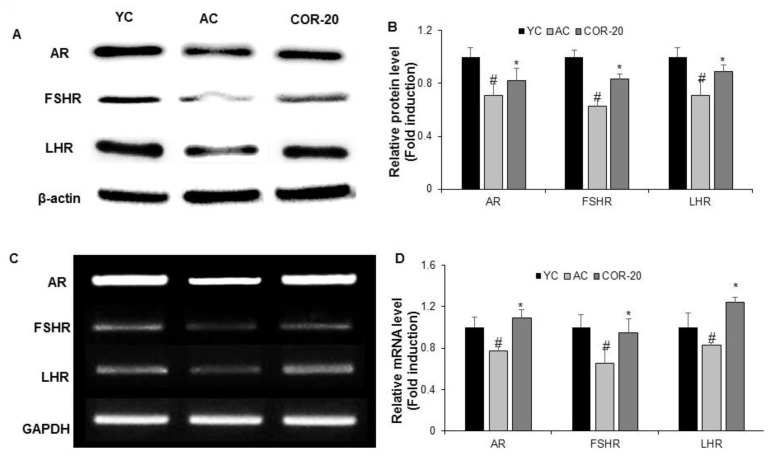
Effect of COR on the expression levels of sex hormone receptors. (**A**) Protein expression of AR, FSHR, and LHR. (**B**) Relative expression levels (fold) in three independent experiments normalized to β-actin. (**C**) The mRNA expression of AR, FSHR, and LHR. (**D**) Relative expression levels (fold) in three independent experiments normalized to that of GAPDH. ^#^ *p* < 0.05 compared with YC group and ^*^ *p* < 0.05 compared with the AC group. AR, androgen receptor; LHR, luteinizing hormone receptor; FSHR, follicle-stimulating hormone receptor; YC, young rat group; AC, aged rat group; COR-20, cordycepin (20 mg/kg) treated AC group; GAPDH, glyceraldehyde 3-phosphate dehydrogenase.

The protein and mRNA levels of androgen receptor (AR), FSH receptor (FSHR), and LH receptor (LHR) and their corresponding quantification are shown in Figure 3. The protein levels of AR, FSHR, and LHR in the AC group were significantly lower (*p* < 0.05) than those in the YC group (Figure 3A,B). In contrast, the protein levels of the above sex hormone receptors were significantly higher in the COR-20 group (*p* < 0.05) than in the AC group. A similar pattern was observed with the corresponding mRNA expression levels. COR 20 mg/kg treatment significantly (*p* < 0.05) attenuated the reduction in the mRNA levels of receptors in the AC group (Figure 3C,D).

### 3.5. Effect on the Expression of Antioxidant Enzymes in Aged Rat Testes

**Figure 4:**
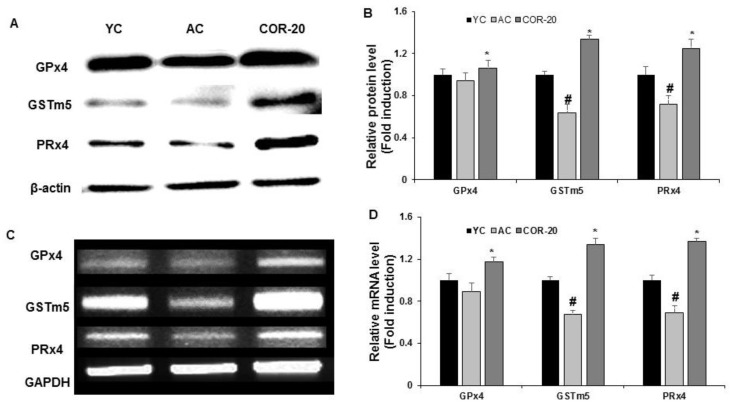
Effect of COR on expression levels of antioxidant enzymes in rat testes. (**A**) Protein expression of GPx4, GSTm5, and PRx4. (**B**) Relative expression levels (fold) in three independent experiments normalized to β-actin. (**C**) The mRNA expression of GPx4, GSTm5, and PRx4. (**D**) Relative expression levels (fold) in three independent experiments normalized to that of GAPDH. ^#^ *p* < 0.05 compared with YC group and ^*^ *p* < 0.05 compared with AC group. YC, young rat group; AC, aged group; COR-20, cordycepin (20 mg/kg) treated AC group; PRx4, peroxiredoxin-4; GSTm5, glutathione S-transferase mu 5; GPx4, glutathione peroxidase 4; GAPDH, glyceraldehyde 3-phosphate dehydrogenase.

To evaluate the effect of COR on the testicular oxidation status in aged rats, the expression of key antioxidative enzymes, such as glutathione peroxidase (GPx4), glutathione S-transferase mu 5 (GSTm5), and peroxiredoxin (PRx4), was analyzed. As shown in Figure 4, the protein levels of these enzymes in the AC group were lower than those with the YC group. Quantification revealed a significant decrease (*p* < 0.05) in GSTm5 and PRx4 levels. Although not significant, GPx4 expression in the AC group was lower than that in the YC group (Figure 4A,B). A similar pattern was observed in the mRNA expression levels, with the levels in the COR-20-treated group significantly (*p* < 0.05) higher than those in the AC group (Figure 4C,D). 

### 3.6. Effect on Spermatogenesis-Related Transcription Factors Expression in Aged Rat Testes

**Figure 5:**
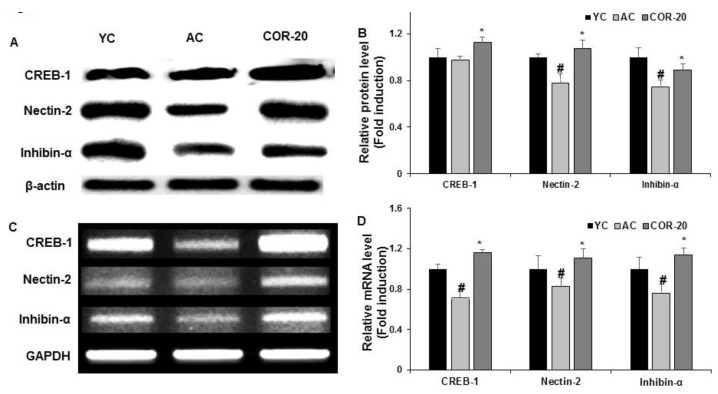
Effect of COR on expression of key biomolecules involved in spermatogenesis. (**A**) Protein expression of CREB-1, nectin-2, and inhibin-α. (**B**) Relative expression levels (fold) in three independent experiments normalized to β-actin. (**C**) The mRNA expression of CREB-1, nectin-2, and inhibin-α. (**D**) Relative expression levels (fold) in three independent experiments normalized to that of GAPDH. ^#^ *p* < 0.05 compared with YC group and ^*^ *p* < 0.05 compared with the AC group. YC, young rat group; AC, aged rat group; COR-20, cordycepin (20 mg/kg) treated AC group; CREB-1, cyclic adenosine monophosphate (cAMP) responsive element binding protein; GAPDH, glyceraldehyde 3-phosphate dehydrogenase.

The expression of molecules involved in spermatogenesis, such as cyclic adenosine monophosphate (cAMP) response-element binding protein-1 (CREB-1), nectin-2, and inhibin-α, is shown in Figure 5. The AC group showed lower protein and mRNA expression levels than the YC group (Figure 5A,C). Quantification of the results showed that COR 20 mg/kg treatment significantly (*p* < 0.05) restored the protein and mRNA levels of the tested key biomolecules compared with in the AC group (Figure 5B,D). 

### 3.7. Effect on mTORC1 and SIRT1 Expression in Aged Rat Testes

**Figure 6:**
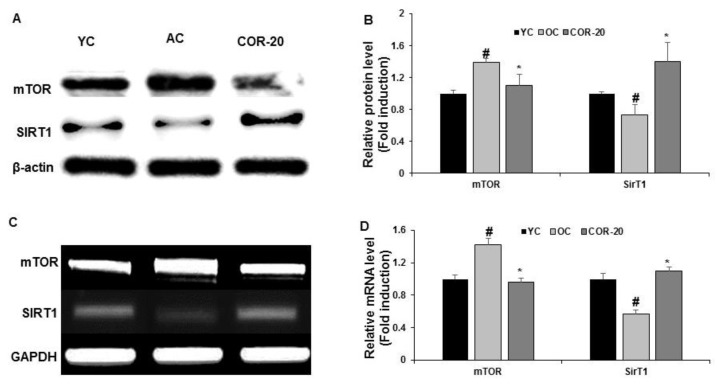
Effect of COR on protein expression levels of mTOR and SIRT1in testis tissue. (**A**) Protein expression of mTOR and SIRT1. (**B**) Relative expression levels (fold) in three independent experiments normalized to β-actin. (**C**) The mRNA expression of mTOR and SIRT1. (**D**) Relative expression levels (fold) in three independent experiments normalized to GAPDH. ^#^ *p* < 0.05, compared with YC group and ^*^ *p* < 0.05 compared with the AC group. YC, young rat group; AC, aged rat group; COR-20, cordycepin (20 mg/kg) treated AC group; mTOR, growth-related mammalian target of rapamycin; SIRT1, silent mating type information regulation 2 homolog; GAPDH, glyceraldehyde 3-phosphate dehydrogenase.

Several reports have revealed that mTORC1 and SIRT1 are key modulators of aging and age-related male reproductive health disorders, including testicular degeneration and abnormal sperm production [29,30]. Therefore, mTORC1 and SIRT1 expression was evaluated to understand the effect of COR on these key molecules. As shown in Figure 6, the protein and mRNA levels of mTORC1 were higher, while those of SIRT1 were lower in the AC group than in the YC group (Figure 6A,C). However, the altered expression was restored in the COR-20 group. Quantification of the results revealed that, compared with the AC group, the COR-20 group showed a significant (*p* < 0.05) reversal of the changes in mTORC1 and SIRT1 levels (Figure 6B,D). 

## 4. Discussion

In the present study, the beneficial effects of COR and its mode of action on age-related sexual dysfunction was confirmed in experimental rat model. It is well known that age-associated alterations in testicular function are based on several parameters, including sperm motility, Sertoli cell number, sperm count in seminiferous tubules, and histopathological examination. Several studies have suggested that aging is associated with decreases in semen parameters, such as motility, sperm count, and Sertoli cell number [31,32]. Aging is involved in the degeneration of germ cells and Sertoli cells, reduction in sperm production, immaturity of spermatozoa, and decrease in seminiferous tubular size. Furthermore, histomorphological examination of the tubular cross sections of aged rats confirmed deficient spermatogenesis [1,33]. In agreement with the results of previous studies, our study showed reduced sperm motility, decreased seminiferous tubule size, and loss of sperm and Sertoli cell count per tubule in the AC group. However, COR treatment effectively ameliorated the age-related changes in histological parameters, such as the Johnsen’s score, seminiferous tubular size, and percentage of tubules containing sperms, sperm count, tubule and germ cell count. These changes were considerably improved by COR treatment. 

To understand the cellular mechanism via which COR exhibits its beneficial effect on aging-induced testicular dysfunction, the protein and mRNA levels of spermatogenesis-related biomarker molecules were investigated. Reproductive function is regulated by sex steroids via their nuclear receptors in the hypothalamus and preoptic area. Studies have demonstrated a relationship between sex hormone receptors and aging-related testicular dysfunction [34]. Sex hormone receptors, such as AR, LHR, and FSHR, play significant roles in maintaining male sexual function, and their altered expression may be involved in aging-related testicular disorders. Additionally, qualitative and quantitative changes in AR, the primary targets of androgenic steroids, as well as mutations in the gene encoding AR disrupt receptor sensitivity, leading to male reproductive system disorders [35]. A reduction in the cytosolic AR population was detected in the hypothalamus of aged castrated rats [36]. Furthermore, LHR and FSHR play crucial roles in the proper functioning of the hypothalamus–pituitary–testicular axis. LHR is necessary for testosterone production in Leydig cells, and cell-specific FSHR expression is involved in the response of Sertoli cells to FSH during spermatogenesis [37]. In our study, we observed significant downregulation of AR, LHR, and FSHR in the testes of aged rats, which was significantly ameliorated by COR, suggesting that 20 mg/kg COR protects rats’ Leydig and Sertoli cells from aging-induced testicular dysfunction.

Key biomolecules such as inhibin-α, CREB-1, and nectin-2 are involved in testicular function and act as major spermatogenic factors [38,39,40]. CREB-1, expressed during the mitotic phase of spermatocytogenesis and the differentiation phase of spermiogenesis, plays multiple roles in testicular development and function [38,41]. CREB-1 may also regulate certain aging-related genes, such as the ataxia–telangiectasia mutated gene [42]. Therefore, CREB-1 may be a key molecular regulator of testicular development and adult spermatogenesis [43]. Nectin-2 is an important adhesion molecule present in the Sertoli–germ cell junction and is required for the maturation of spermatozoa in the seminiferous epithelium [44]. Inhibin-α is an important biomarker of Sertoli cell activity in animals with impaired spermatogenesis, which negatively regulates FSH secretion from the anterior pituitary gland to maintain FSH homeostasis [45]. In the present study, aged rats showed a significant reduction in the expression of inhibin-α, nectin-2, and CREB-1 at both the protein and mRNA levels compared with the YC rats, and this effect was significantly attenuated by treatment with 20 mg/kg COR (*p* < 0.05). This indicates that aging significantly alters the expression of spermatogenesis-related molecules and that COR effectively restores these changes.

Previous reports have indicated that aging involves the accumulation of oxidative damage in cells and tissues and that oxidative stress is the main factor contributing to decreased organ and cell function during aging [8]. High levels of polyunsaturated fatty acids accumulate in mammalian testes with aging, which are prone to free radical attack, creating an oxidative imbalance that results in impairment of steroidal testicular function [9]. Previous reports from our group suggested that COR significantly attenuated age-related oxidative stress and decreased lipid peroxidation in aged rats [27]. PRx4, an antioxidant enzyme that prevents oxidative stress-induced cell damage, is ubiquitously distributed in the mitochondrial matrix and expressed at high levels in reproductive organs [46]. The levels of GST and its GSTm5 form, which occur in fibrous sheaths and promote the proliferation and differentiation of germ cells, may be altered during oxidative stress to combat attack by free radicals in the testes and spermatogenic cells [47,48]. Similarly, GPx4, an antioxidant that protects against free radical-mediated damage to membrane lipids, proteins, and nucleic acids, has also been implicated as an important structural molecule in sperm maturation [49]. Based on these reports, we evaluated the expression of key enzymes that regulate oxidation and play important roles in sperm function and spermatogenesis, such as GPx4, GSTm5, and PRx4. Consistent with the reported results, we observed that the levels of GPx4, GSTm5, and PRx4 in aged rats were downregulated than those in young control rats. COR (20 mg/kg) treatment of aged rats significantly ameliorated the reduction in protein and mRNA levels in the testis tissues (*p* < 0.05). 

The role of SIRT1 in spermatogenesis, including spermatogenic stem cells, as well as germ cell function has been extensively studied [50]. Sirtuins are associated with longevity and are involved in regulating male germ cell lifespan and aging-related decline in sperm quality [30]. In contrast, the levels of mTORC1, a key modulator of autophagy- and age-related diseases, increases when food is abundant, prompting cells to increase their overall protein synthesis and proliferate. mTORC1 level decreases during unfavorable conditions, which reduces the overall production of proteins required for various biological activities, including events that are pivotal for spermatogenesis and the reproductive potential of males [51]. Evidence suggests that mTORC1 regulates male fertility, and its inhibition is currently being investigated for the treatment of certain diseases, such as cancers and defects in male reproduction. Therefore, SIRT1 and mTORC1 can be used as biomarkers of aging and aging-related male reproductive conditions. We observed that the protein and mRNA levels of SIRT1 and p-mTORC1 in the AC group were significantly altered compared with those in the YC group (*p* < 0.05) and that COR treatment (20 mg/kg) attenuated these changes in aged rats, suggesting that COR regulates and delays testicular aging. However, further studies are needed to clearly elucidate the detailed cellular mechanism via which COR improves testicular dysfunction associated with aging.

## 5. Conclusions

Our results indicate that COR potently recovers the biological and physiological feedback mechanism related to sex hormone balance, leading to completion of the spermatogenic process via which mature spermatozoa are produced from spermatogonia in the testes. From the perspective of cellular mechanism, COR appears to be involved in ameliorating aging-induced gene expression associated with testicular function, particularly those related to spermatogenesis, antioxidant defense, acetylation (SIRT1), and autophagy-related (m-TORC1) activity. In conclusion, the present study suggests that cordycepin may provide an excellent nutritional potential and medicinal value in ameliorating age-related male sexual dysfunction. 

## Figures and Tables

**Table 1:** Primers used in study.

Peroxiredoxin (PRx4)	Forward: 5′-CTG ACT GAC TAT CGT GGG AAA TAC T-3′ Reverse: 5′-GAT CTG GGA TTA TTG TTT CAC TAC C-3′
Glutathione S-transferase mu 5 (GSTm5)	Forward: 5′-TAT GCT CCT GGA GTT TAC TGA TAC C-3′ Reverse: 5′-AGA CGT CAT AAG TGA GAA AAT CCA C-3′
Glutathione peroxidase (GPx4)	Forward: 5′-GCA AAA CCG ACG TAA ACT ACA CT-3′ Reverse: 5′-CGT TCT TAT CAA TGA GAA ACT TGG T-3′
Inhibin-α	Forward: 5′-AGG AAG GCC TCT TCA CTT ATG TAT T-3′ Reverse: 5′-CTC TTG GAA GGA GAT ATT GAG AGC-3′
Androgen receptor (AR)	Forward: 5′-CTG GAC TAC CTG GAT CTC TA-3′ Reverse: 5′-CCT GGG CTG TAG TTT TAT TG-3′
Follicle-stimulating hormone receptor (FSHR)	Forward: 5′-GGA CTG AGT TTT GAA AGT GT-3′ Reverse: 5′-TTC CAT AAC TGG GTT CAT CA-3′
Luteinizing hormone receptor (LHR)	Forward: 5′-CTA TCT CCC TGT CAA AGT AA-3′ Reverse: 5′-TTT GTA CTT CTT CAA ATC CA-3′
Nectin-2	Forward: 5′-AGT GAC CTG GCT CAG AGT CA-3′ Reverse: 5′-TAG GTA CCA GTT GTC ATC AT-3′
Glyceraldehyde-3-phosphate dehydrogenase (GAPDH)	Forward: 5′-AAC TTT GGC ATT GTG GAA GGG C-3′ Reverse: 5′-ACA CAT TGG GGG TAG GAA CAC G-3′
Cyclic adenosine monophosphate (cAMP) response-element binding protein-1 (CREB-1)	Forward: 5′-ACT GGC TTG GCA CAA CCA GA-3′ Reverse: 5′- GGC AGA AGT CTC TTC ATG ATT-3′
Mammalian target of rapamycin complex 1 (mTORC1)	Forward: 5′-GACAACAGCCAGGGCCGCAT-3′ Reverse: 5′-ACGCTGCCTTTCTCGACGGC-3′
Sirtuin 1 (SIRT-1)	Forward: 5′-GCTGGGGTTTCTGTCTCCTG-3′ Reverse: 5′-GACACAGAGACGGCTGGAAC-3′

**Table 2:** Effect of cordycepin on sperm kinematics.

Parameters	YC	AC	COR-5	COR-10	COR-20
Motility (%)	74.70 ± 8.80	34.60 ± 8.70^#^	40.40 ± 18.40	59.70 ± 6.50^*^	67.00 ± 5.90^**^
Progressive (%)	33.70 ± 4.20	12.70 ± 3.90^#^	15.40 ± 7.40	22.20 ± 2.20^*^	29.20 ± 2.10^**^
VAP (µM/s)	220.30 ± 32.80	155.30 ± 14.60^#^	179.90 ± 17.60	188.00 ± 11.40^*^	209.70 ± 9.30^**^
VSL (µM/s)	158.20 ±27.50	113.40 ± 12.60^#^	129.10 ± 12.20	134.30 ± 13.40^*^	145.10 ± 21.50^**^
VCL (µM/s)	268.30 ± 25.50	270.50 ± 80.70	265.50 ± 38.90	278.70 ± 28.70	334.80 ± 51.20
LIN (%)	58.70 ± 6.20	32.70 ± 9.50^#^	34.40 ± 1.90	38.70 ± 0.70	45.60 ± 1.60^*^
STR (%)	71.50 ± 4.30	72.50± 11.90	71.50 ± 1.20	71.80 ± 0.80	69.10± 2.30
WOB (%)	81.90 ± 6.70	59.80 ± 11.80^#^	63.70 ± 1.60	68.30 ± 0.70^*^	72.70 ± 1.10^**^

^#^*p* < 0.05 compared with YC group; ^*^ *p* < 0.05 and ^**^ *p* < 0.01 compared with the AC group. COR-5, cordycepin 5 mg/kg; COR-10, cordycepin 10 mg/kg; COR-20, cordycepin 20 mg/kg; YC, young control; AC, aged control; LIN, linearity (VSL/VCL × 100); SEM, standard error of the mean; STR, straightness (VSL/VAP×100); VAP, average path velocity; VCL, curvilinear velocity; VSL, straight line velocity; VAP, average path velocity; WOB, wobble (VAP/VCL × 100).

## Supplementary Materials

The following are available online at https://www.mdpi.com/2072-6643/11/4/906/s1, Figure S1: HPLC chromatogram of cordycepin. Instrument, Waters HPLC system; Column, YMC PacK ODS (4.6 X 250 mm); mobile phase, 14% acetonitrile; flow rate, 0.8 mL/min; detection, UV 260 nm.
