# Supplementary material for: Cordycepin, an Active Constituent of Nutrient Powerhouse and Potential Medicinal Mushroom Cordyceps militaris Linn., Ameliorates Age-Related Testicular Dysfunction in Rats
Source: Nutrients. 2019 Apr 23;11(4):906. doi: 10.3390/nu11040906 (PMC6520895; doi:10.3390/nu11040906)
Supplement: Supplementary file 1 [file nutrients-11-00906-s001.pdf]

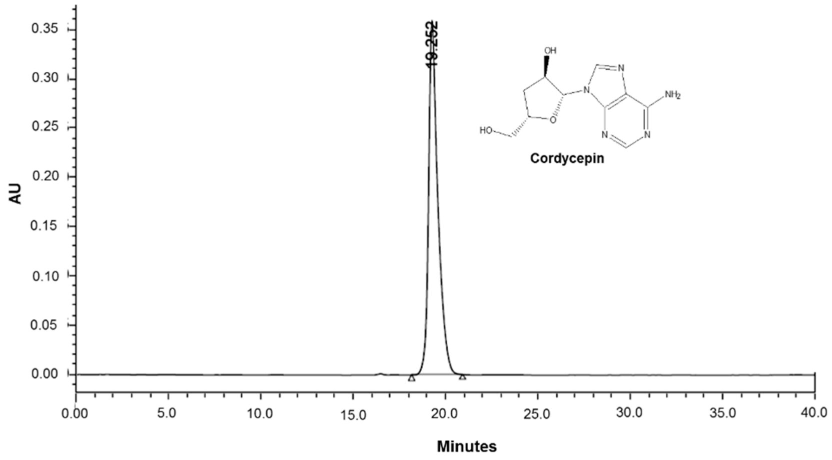

Figure S1. HPLC chromatogram of cordycepin. Instrument; Waters HPLC system, Column; YMC PacK ODS (4.6 X 250 mm), Mobile phase; 14% acetonitrile; Flow rate; 0.8 mL/min, Detection; UV 260 nm.
